# Tissue-Preserving Inframammary Fold Breast Augmentation: A 3-Year Clinical Experience

**DOI:** 10.1093/asj/sjag038

**Published:** 2026-02-06

**Authors:** Charles Randquist, Jessica Gahm, Apinut Wongkietkachorn, Marie Jaeger

## Abstract

**Background:**

Breast surgery often compromises structures, potentially leading to complications that affect aesthetic outcomes, lactation, breast tissue health, and the patient experience. Tissue-Preserving Breast Augmentation (TPBA) is a novel concept designed to enhance breast and implant stability by preserving the breast's stabilization system, including the circummammary ligament and Cooper's suspensory ligaments.

**Objectives:**

This study presents the TPBA approach, performed in a minimally invasive manner to minimize surgical stimuli and protect the parenchymal breast tissue, along with outcomes observed over a 3-year period.

**Methods:**

This 2-center, multi-surgeon retrospective observational study included consecutive female patients undergoing primary breast augmentation or primary augmentation–mastopexy using TPBA between December 2022 and June 2025. All procedures involved an inframammary incision and creation of a prepectoral pocket through balloon expansion. Complications were assessed by monitoring cumulative incidence during follow-up visits at 2 weeks, 6 months, 12 months, and 24 months.

**Results:**

A total of 330 procedures were performed. At a mean follow-up of 18 months, the complication rate was 0.9%. No cases of acute complications, including seroma, wound breakdown, or infection, were observed. Follow-up evaluations revealed no capsular contracture (Grades III and IV), implant rupture, lateralization, or inferior malposition. Superior malposition occurred in 2 patients (0.6%), and bleeding was observed in 1 patient (0.3%). Implant rippling was reported by 3 patients (0.9%).

**Conclusions:**

TPBA offers a standardized, reproducible approach to breast enhancement through the inframammary fold, performed under general or local anesthesia. It preserves stabilization structures, features a short learning curve, and carries a low risk of complications.

**Level of Evidence: 4 (Therapeutic):**

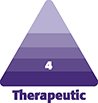

Since the introduction of breast implants in the early 1960s, numerous approaches have been proposed to enhance surgical techniques in both aesthetic and reconstructive breast surgery.^1^ Similarly, in recent decades, there have been numerous efforts to improve implant characteristics and prevent prevalent complications.^1^ Among these developments is the development of various implant surface technologies.

Despite the existence of an updated classification norm for breast implants based on surface roughness, which categorizes them into 3 main types: nonintentionally textured surfaces, implants with additional surface coatings (such as polyurethane), and intentionally textured silicone shells, further distinctions are made within the textured category.^2^ These are classified into microtextured surfaces (<50 μm) and macrotextured surfaces (>50 μm). This classification acknowledges that a range of clinical outcomes may occur within each category.^2^ Therefore, from both clinical and surgical perspectives, and in alignment with the most recent literature, it is important to examine the factors relevant to the surgical approach discussed in this paper by incorporating the previous classifications, as well as the evolving experience of surgeons over time.

Textured implants (commonly defined by surface topography as having an average roughness >10 μm) are recognized for a low incidence of malposition complications over time, such as lateralization and inferior malposition.^3,4^ They also demonstrate a reduced risk of capsular contracture, especially when placed in a subglandular pocket.^5^ However, these implants are known to induce biofilm formation.^6-8^ Because of their association with an increased risk of breast implant–associated anaplastic large-cell lymphoma (BIA-ALCL), there has been a notable shift toward the use of smooth-surfaced implants.^9-12^ Some textured implants have been banned in certain countries, whereas others have been voluntarily withdrawn from the market.^13,14^ As a result, surgeons who were previously technically familiar with textured implant surfaces may now be navigating the unfamiliar behavior of smooth-surfaced implants.

Smooth implants (commonly defined by surface topography as having an average roughness of <10 μm) do not seem to be associated with an increased risk of BIA-ALCL and exhibit reduced bacterial biofilm formation.^3,6,8,9-11^ Historically, traditional smooth implants have been stigmatized with a higher risk of malposition and capsular contracture, the latter one, especially when placed in a subglandular pocket, which, paradoxically, in certain cases might have stabilized the implant position, depending on the level of capsular formation, like an inner bra, thereby reducing the likelihood of malposition.^4,5,15^

Recent studies indicate that implants featuring a more biocompatible, 4-micron surface result in thinner capsule formation and reduced rate of capsular contracture compared with previously published studies that combined data across various surface types.^5,16-19^ This improvement is thought to reflect a decreased body immune response.^6,15,16^ However, this reduced inflammatory tissue formation and thinner capsule formation associated with the 4-micron surface may lead to a more unpredictable tissue stretch over time, influenced by the interplay between implant weight, breast tissue integrity, and skin quality. Therefore, there is a potential long-term risk of complications, such as inferior malposition, implant lateralization, inframammary fold (IMF) breakdown, tissue atrophy, pain, visible implant edges, and rippling.

In 1996, the first author (C.R.) began predominantly using anatomical textured implants, along with the principles of sharp submuscular, dual-plane pocket dissection performed under direct vision.^20^ In 2015, the SmoothSilk implants (Establishment Labs Holdings, Inc., Alajuela, Costa Rica), with an average roughness of 4 microns, were incorporated into the clinical practice at Victoriakliniken, applying these previously established surgical principles.^16^ However, in 2018, these principles were revised in response to several cases of inferior malposition and lateralization with SmoothSilk implants. A more stringent patient and implant selection process was adopted, shifting to the use of smaller, less projected SmoothSilk implants placed in a prepectoral position, directly on top of the fascia. This strategy aimed to avoid the influence of muscle forces that could expand the pocket over time. This shift to a tighter, under-dissected, and less invasive prepectoral pocket improved implant stability and prevented potential movements and malpositioning of the implant, as previously documented.^17,18,21^

Historically, despite numerous efforts and approaches, there has been little emphasis on appreciating the breast anatomy and safeguarding its essential structures during breast surgeries.^22^ The anatomy of the female breast is intricate and plays a crucial role for both lactation and overall bodily function, while also contributing to a patient's perception of physical attractiveness and femininity.^23,24^

## Superficial Fascia System

Understanding the Tissue-Preserving Breast Augmentation (TPBA) technique lies in appreciating the crucial role of the SFS in maintaining breast shape and stability. This 3-dimensional network, comprised of fat and fascia, forms a structural bridge between the superficial layer (skin) and deep-layered structures (underlying chest wall).^25^

**Figure 1.**
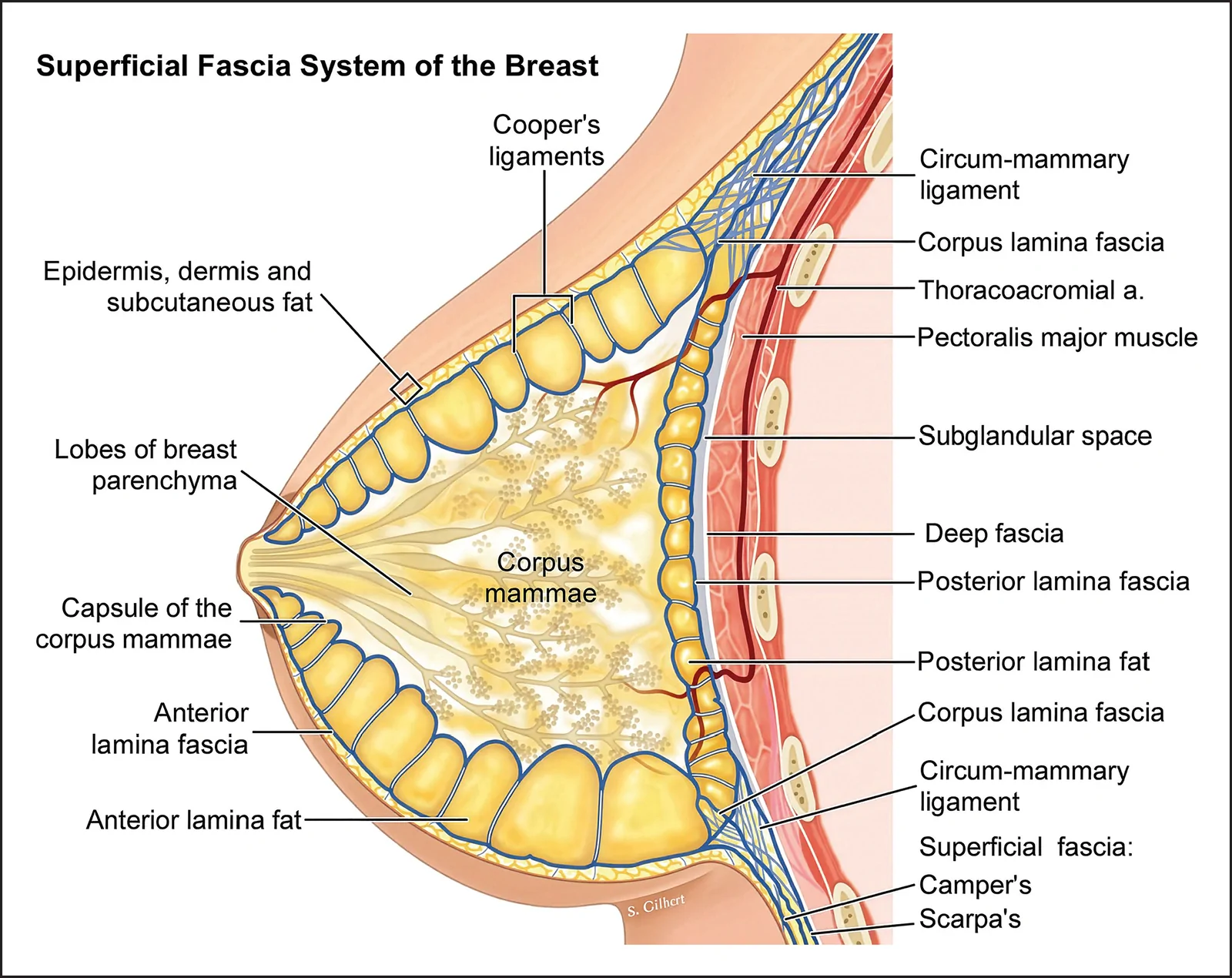
Superficial fascia system of the breast. Illustration created by Dr Susan Gilbert, medical illustrator. Originally published in: Rehnke RD, Clarke JM, Goodrum AJ, Badylak SF. Absorbable Biosynthetic Scaffolds in Place of Silicone for Breast Reconstruction: A 9-Year Experience with 53 Patients. *Plast Reconstr Surg Glob Open.* 2024;12(5):e5821, reproduced with permission from Wolters Kluwer Health, Inc. (Philadelphia, PA).^25^

The corpus mammae is situated between 2 layers of the superficial fascia, separated by a layer of fat that defines the anterior and posterior lamellae, each with its respective fascia.^25^ Among the most prominent anatomical features in breast surgery, the Cooper's ligaments run vertically from the posterior fascia, through the breast gland and anterior lamella, ultimately anchoring into the skin (Figure 1).^26^

## Circummammary Ligament

**Figure 2.**
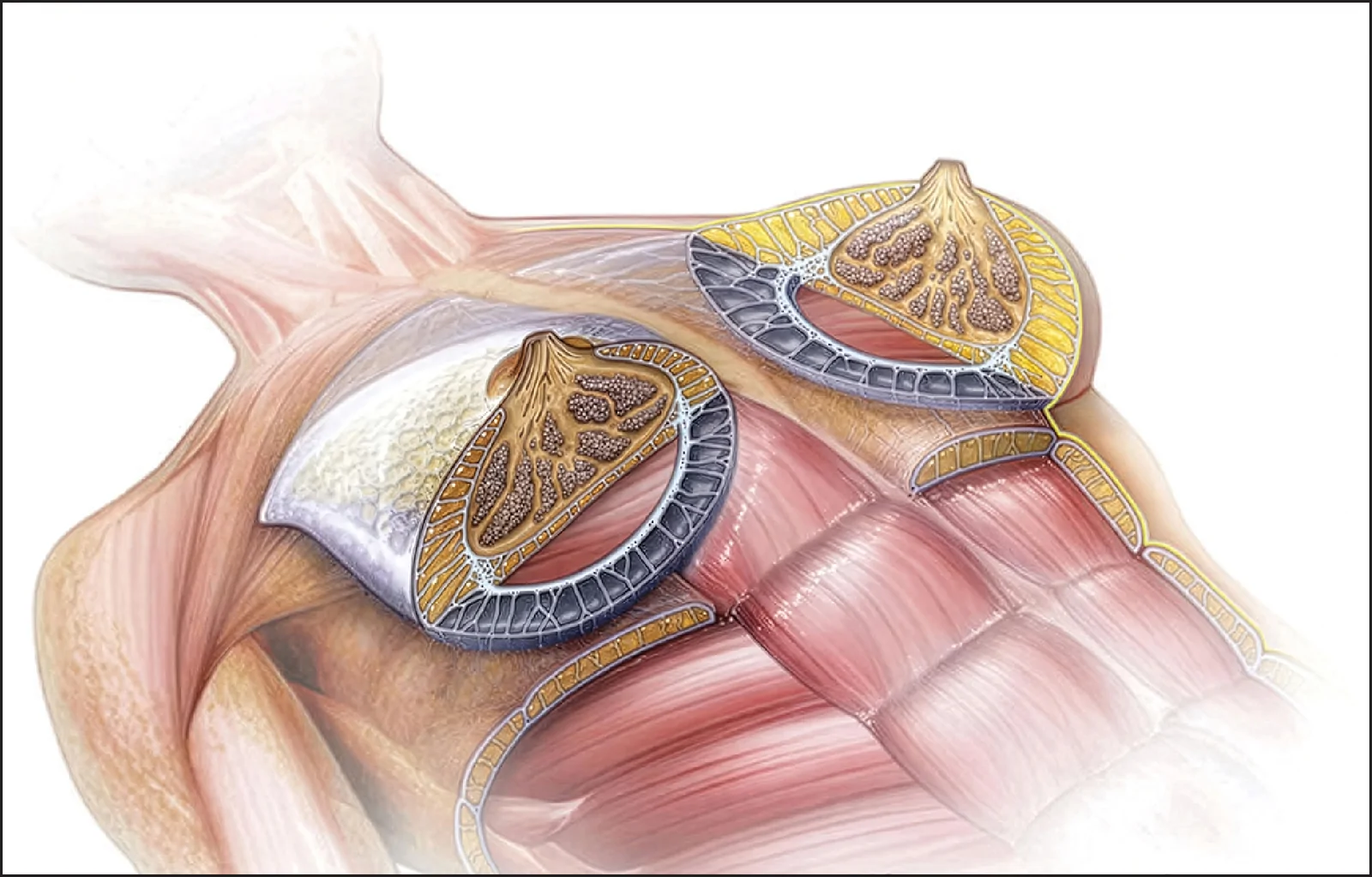
Superficial fascial system with the circummammary ligament encircling the base of the breast. Created by Illumination Studios (Dallas, TX).^25^ Originally published in: Rehnke RD, Groening RM, Van Buskirk ER, Clarke JM. *Plast Reconstr Surg.* 2018;142(5):1135-1144, reproduced with permission from Wolters Kluwer Health, Inc. (Philadelphia, PA).^25^

First described by Rehnke et al, the circummammary ligament (CML) is identified as a key structural element in breast anatomy.^25^ This fibrous condensation of connective tissue represents the fusion of the anterior and posterior lamellae of the superficial fascia with the deep fascia of the chest.^25^ The CML encircles the base of the breast and demarcates the natural transition between the breast parenchyma and the chest wall (Figure 2).^25^

Functionally, the CML provides additional support to the breast footprint, helps define the borders of the breast mound, and contributes to the stability of the IMF. Its inferior and inferolateral portions anchor to the chest between the fifth rib and sixth intercostal space.^25^ Preservation of this structure is therefore critical to maintaining the natural breast envelope, preventing implant malposition, and ensuring long-term aesthetic outcomes. Additionally, this ligament serves as a fixed conduit for nerves, lymphatics, and blood vessels.^25^

These supportive structures may be affected by natural factors such as aging, weight fluctuations, pregnancy, lactation, and genetic predisposition. Additionally, they are often damaged by tissue trauma during breast surgery, which subsequently weakens the structural integrity of the breast. Procedures such as reduction mammoplasty, mastopexy, and augmentation often compromise the internal breast structures through extensive tissue manipulation, long incisions, or minimal focus on tissue preservation. Such disruptions may result in postoperative complications, including implant malposition, capsular contracture, breast asymmetry, contour irregularities, double-bubble deformity, ptosis, loss of nipple sensation, and diminished aesthetic appearance of the breast, in addition to lactation capabilities and tissue health, ultimately affecting the overall patient experience.^27^

Although preserved but stretched, the authors observed that these 3-dimensional structures could provide stability for prepectoral-positioned breast implants. Therefore, adopting a surgical approach that prioritizes tissue preservation during breast augmentation is critical for achieving both aesthetic and functional success. By employing an atraumatic, blunt dissection technique, the native anatomical structures and borders of the breast, including the IMF, are not disrupted, except for a short incision made in the IMF. The first atraumatic TPBA procedure was performed under local anesthesia at Victoriakliniken in June 2021 by the senior author, C.R.

In this technical paper, the authors describe their experience with TPBA, performed using the prepectoral plane and an IMF approach. TPBA is a novel concept that aims to enhance breast and implant stability by preserving the breast's natural stabilization system, specifically the superficial fascia system (SFS). The procedure is performed in a minimally invasive manner to prevent extensive invasive surgical stimuli and avoid unnecessary damage to the breast tissue. Additionally, TPBA promotes several clinical advantages: it can be performed under local anesthesia or in combination with light sedation, incorporates bolus hydrodissection, and thereby reduces both operative and recovery time, as well as the risks associated with conventional dissection techniques and general anesthesia.

## METHODS

This retrospective, multisurgeon observational study was conducted across 2 centers and included all consecutive female patients meeting inclusion criteria during the study period and undergoing breast augmentation using the TPBA technique between December 2022 and June 2025. The cohort included both primary breast augmentation and primary augmentation–mastopexy cases. Secondary or revision procedures were excluded. All procedures were performed by the 4 senior authors; 3 (C.R., J.G., and M.J.) are board-certified plastic surgeons practicing at Victoriakliniken in Stockholm, Sweden, whereas the fourth (A.W.) is a board-certified plastic surgeon at Clinic One in Bangkok, Thailand. A.W. received training in the TPBA technique during a fellowship at Victoriakliniken in 2022. IRB approval was not required, because this study represents a retrospective analysis of standard clinical practice without experimental intervention. All patients provided written informed consent for surgery and for inclusion of anonymized data and images for research and publication. The study was conducted in accordance with the Declaration of Helsinki. The majority of procedures were performed under local anesthesia with or without light sedation, whereas all augmentation–mastopexy procedures were performed under general anesthesia.

### Surgical Planning

The midline (on top of the sternum) and a vertical line 2 cm on each side of the midline are marked to create a 4 cm no-touch zone, intended to avoid disruption of medial breast stability that could lead to implant medialization and increase the risk of symmastia in a prepectoral pocket.^28^ The natural IMF is identified, and a 2.5 to 3.5 cm incision, depending on the size and type of the chosen implant, is marked at the 6 o’clock position below the nipple–areolar complex (NAC). The width of the chosen implant should not exceed the natural breast's width minus 1 cm, to avoid overstretching the tissue beyond its anatomical boundaries. The marking on the breast should be based on the width of the implant minus 1 cm, and with the NAC positioned in the center of the circle as the highest projecting point. This marking serves as a guideline for the prepectoral balloon-assisted pocket creation and ensures a tight pocket dissection (Video 1).

### Medications

Two hours preoperatively, patients are administered orally a single dose of 2 g tranexamic acid, 5 mg oxycodone, 1 g paracetamol, and 25 mg meclizine. During induction, a single intravenous (IV) dose of 2 g cloxacillin is administered; for patients with a penicillin allergy, 600 mg of clindamycin is used.

No pocket irrigation is performed intraoperatively, and no prophylactic antibiotics are given postoperatively. For pain management during the first 2 postoperative days, patients receive 1000 mg paracetamol every 6 h and ibuprofen 400 mg every 8 h. Gabapentin may be considered for patients presenting with neuropathic pain secondary to stretching of ligaments following implant insertion and postoperative swelling.

### Technique

After washing and prepping the surgical site, nipple shields are placed before infiltrating 5 mL of local anesthesia into each IMF incision and down to the periosteum of the underlying rib, followed by 20 mL into each lower breast pole. A 2.5 to 3.5 cm IMF incision is then made centrally at 6 o’clock, followed by blunt scissor dissection through the Scarpa's fascia, down to the fascia of the pectoralis major muscle (PMM). Once the fascia is visualized, the surgeon inserts a blunt injection cannula (2 mm × 20 cm) and carefully advances it cranially along the surface of the PMM fascia until the tip of the cannula is positioned behind the center of the NAC. Hydrodissection is performed using a bolus of 250 mL tumescent solution on each side, effectively separating the (posterior lamella and) breast parenchyma from the PMM fascia. The breast is then massaged to ensure optimal distribution of the fluid (Video 1).

**Figure 3.**
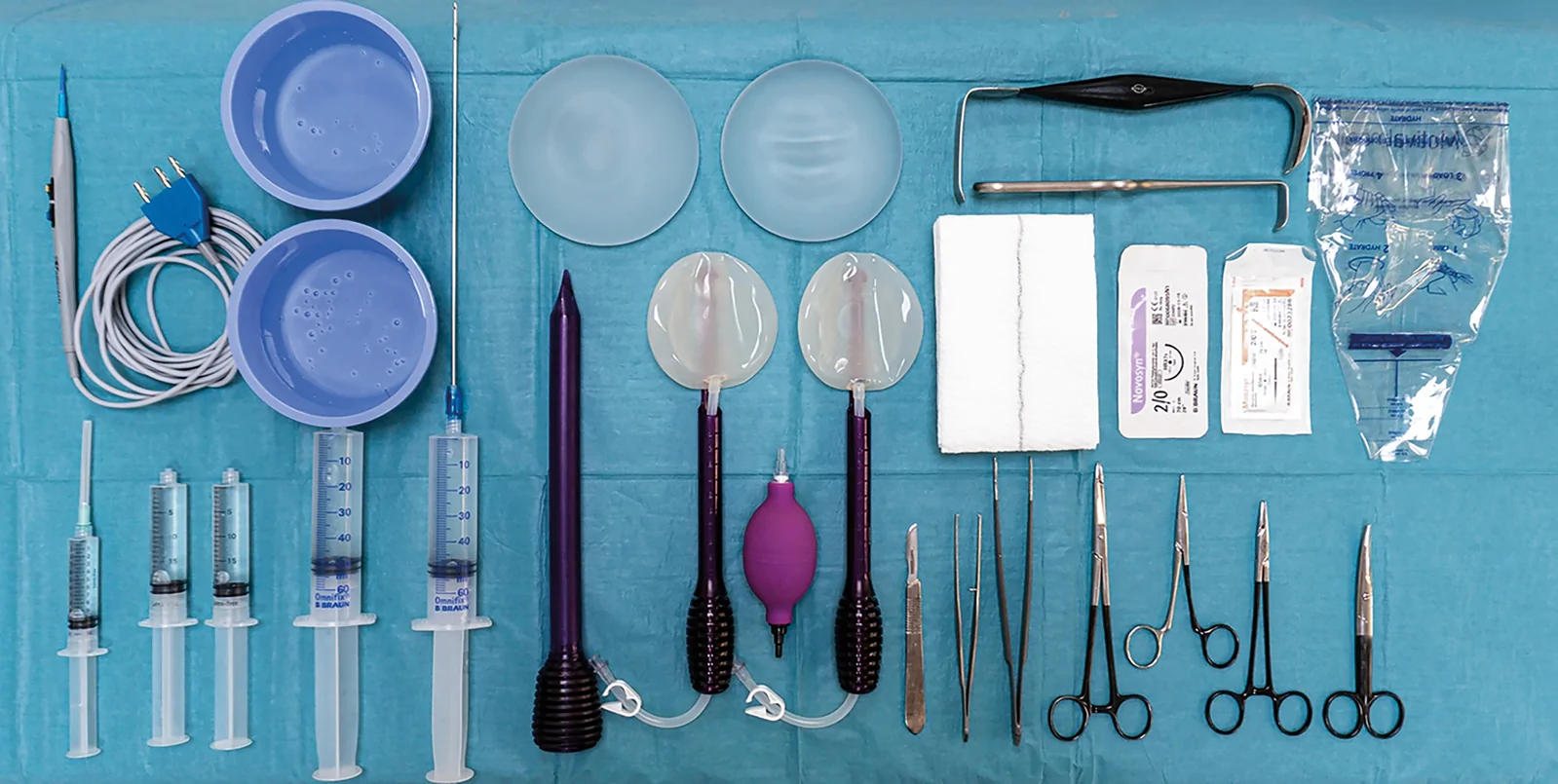
Surgical instrumentarium used to perform the Tissue-Preserving Breast Augmentation technique.

Upon removal of the cannula, following the same plane and direction, a channel separator with a diameter of 2 cm (Establishment Labs) is employed to create a narrow channel on top of the PMM fascia, extending 2 cm beyond the cranial implant markings on the breast, to accommodate the tip of the separator. When reaching the infiltrated area, some of the previously injected fluid drains, confirming correct positioning and dissection plane. At this stage, the prefascial positioning of the channel is verified under direct visualization using a retractor. No lateral, cranial, or medial dissection should be performed to preserve the integrity of the tight channel. The complete surgical instrumentation used to perform the TPBA technique is shown in Figure 3.

A deflated, folded balloon attached to a stable guide (Establishment Labs) is inserted into the channel created in the first breast and advanced to the end of the channel. The inflatable balloon has the same projection as the selected implant but is 1 cm narrower in width, ensuring a tight pocket. Following insertion, the surgical assistant inflates the balloon with air to match the selected implant volume. Throughout the expansion process of the balloon, the plastic surgeon places one hand on the lateral breast and the other on the guide, maintaining the balloon's position behind the NAC to prevent uncontrolled expansion beyond the marked boundaries.

The first balloon is left in place, allowing for pressure-assisted hemostasis, whereas tunnel dissection and pocket expansion are performed on the contralateral side. Once expansion is completed, the shape of the lower pole is evaluated. The lateral, upper, and upper medial poles typically appear round, whereas the lower pole, especially the medial aspect, often exhibits retaining bands that inhibit roundness. This is attributed to the presence of more fibrotic and stronger ligaments in this region.

In such cases, although the balloon remains in position, the guide is removed, and additional blunt finger dissection of the retaining bands may be conducted to finalize the round shape of the prepectoral pocket. Any dissection in the lateral lower pole should be performed with utmost care to avoid implant lateralization. With both balloons in place, asymmetries and final adjustments of the lower pole may now be addressed (Video 2).

The inflatable balloon on the first side is then deflated and removed, and a nonwoven gauze soaked in 50 mL saline and 1 mL (1 mg/mL) epinephrine is placed into the pocket. Hemostasis is thereby maintained through a 2-pronged approach: first, by localized pressure from the balloon, and second, by the profound vasoconstriction of the epinephrine.^29^ After the creation of both prepectoral pockets, the saturated gauze is removed, and a final bleeding control is conducted under direct observation. No pocket irrigation is performed. Endoscopic visualization, as shown in Video 3, is not part of the routine operative workflow and is included for educational purposes only to demonstrate the final, precisely prepared, near-bloodless pocket following balloon expansion.

Before opening the implant boxes, 10 cc of normal saline is injected into each sterile box containing the implants. Presoaking the devices eradicates static electricity, thereby reducing the risk of airborne particle adhesion when the implants are removed from their boxes and exposed to air.^30^ At this stage, the surgeon changes into a new pair of surgical gloves, ensuring that no surfaces are touched except for the saline-activated funnel used for implant insertion. The implant, SmoothSilk Round, Ergonomix, or Ergonomix2 (Motiva, Establishment Labs), is positioned with its posterior side against the backside of the funnel.

The surgeon then performs a final bleeding control under direct visualization using a dry, nonwoven gauze. A narrow retractor gets inserted into the pocket, and special care is taken to avoid lifting the retractor, as this could allow air to enter the pocket before funnel and implant insertion. This precaution prevents air from filling the pocket. Air trapped within the pocket could cause resistance and a more challenging implant insertion because of the possible airlock created by the funnel and implant. Instead, the surgeon inserts the tip of the funnel beyond the incision in an airtight manner, the assistant pushes out the air from the breast pocket, and once the surgeon pushes the implant forward toward the incision, the assistant lifts the retractor, easing the insertion by creating a negative pressure, a method referred to by the authors of this paper as the “FANAI technique” (Funnel Assisted No Airlock Implantation) (Video 3).

**Figure 4.**
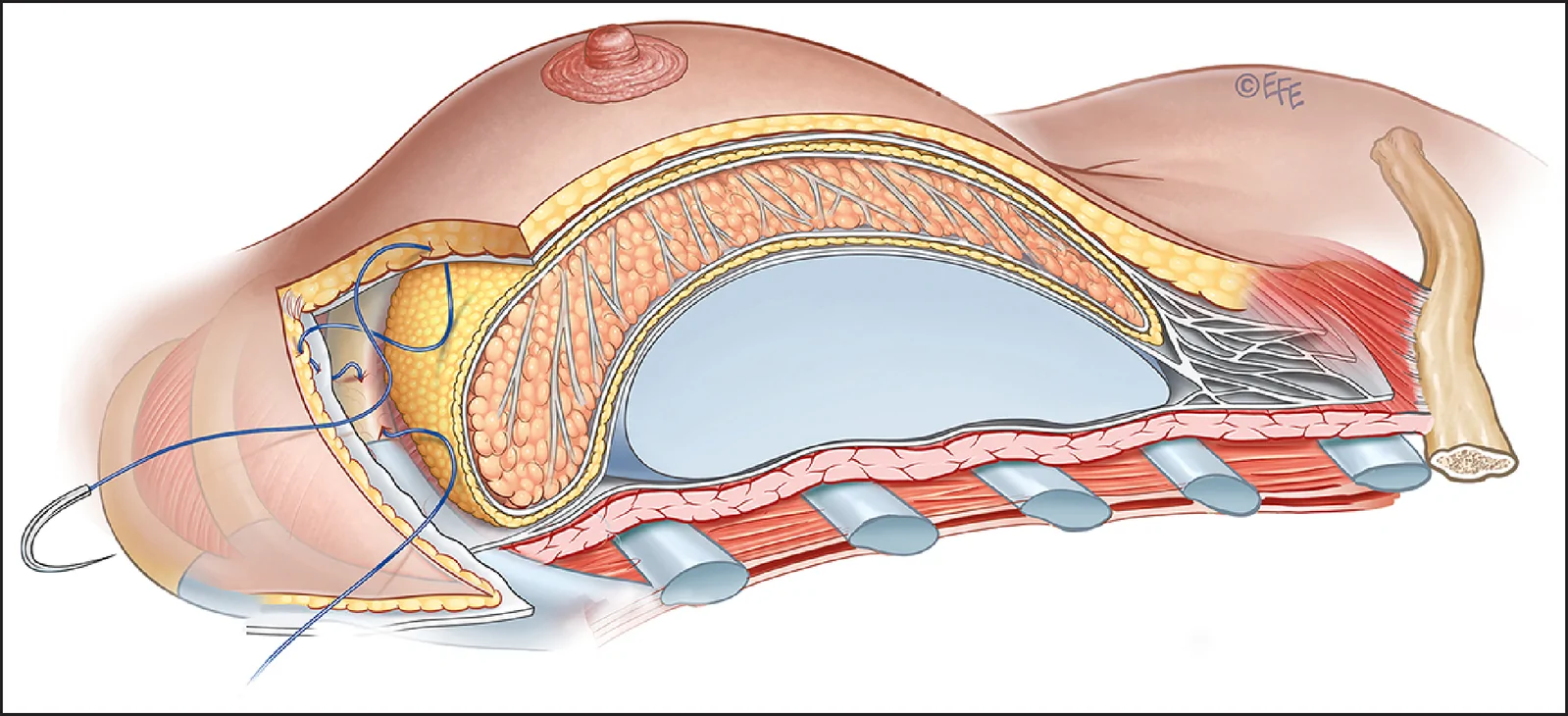
Figure-of-eight suture: Lucky 8 stitch. Illustration created by Dr Levent Efe, medical illustrator.

Fixation of the IMF and the incision into the IMF fold are achieved using a single, 3-point figure-of-eight suture (“Lucky 8 stitch”) with a 2-0 absorbable braided suture featuring a sharp, not-cutting tip (B. Braun; Melsungen, Germany), as shown in Figure 4.^28^ Intradermal skin closure is performed using an absorbable 2-0 monofilament suture, followed by the application of a water-resistant spray plaster and Micropore paper tape (Video 4). In augmentation–mastopexy cases, the TPBA-based augmentation is performed first, followed by the mastopexy; all augmentation–mastopexy procedures in this series were conducted under general anesthesia, with an intentionally reduced use of local anesthetic to limit breast swelling, facilitate accurate skin tailoring, and avoid excessive cumulative local anesthetic dosing.

### Postoperative Care and Follow-Up

Patients get discharged home within 90 min following surgery and can resume their daily activities the same day and light sport activities within 3 weeks. They are informed about expected swelling because of infiltrated fluid and are instructed to wear a breast girdle, along with a 4 cm wide by 10 cm high wound dressing roll placed on the sternum between the breasts for the first week. Additionally, a tight surgical compression bra is recommended to be worn continuously for at least 3 weeks to prevent implant displacement and reduce swelling. Showers are permitted after 3 days, and the dressing tape gets removed during the first follow-up visit, which occurs within 7 to 14 days postoperatively. Patients are advised to contact the clinic in case of any adverse events such as sudden swelling, pain, redness, or fever. For optimal scar healing, silicone tape is recommended starting at 6 weeks postoperatively and for a duration of 6 months. Subsequent ultrasound-assisted follow-up visits are conducted at 6 months, 12 months, 2 years, and then every other year, following a predesigned protocol that includes photographic documentation and measurements.

Complications were assessed by monitoring cumulative incidence during follow-up visits at 2 weeks, 6 months, 12 months, and 24 months postoperatively. The following complications were assessed during these visits: seroma, defined as a sudden change in breast size resulting in asymmetry, capsular contracture (defined as Baker grade III–IV), inferior or lateral implant malposition (referring to implant displacement where the implant either drops below the IMF scar or stretches out the lower pole, leading to a high-riding nipple, or implants that slide laterally toward the armpits), double bubble, characterized by a crease across the lower part of the augmented breast that creates 2 distinct breast mounds, rippling, identified as visible folds in the implant seen through the breast skin, and implant rupture, indicated by a loss of implant resistance or spontaneous regaining of shape upon physical examination.

## RESULTS

A total of 330 consecutive female patients (660 implants) were operated on with the TPBA technique between December 2022 and June 2025. The mean patient age at the time of surgery was 37 years (range, 18-63 years), the mean patient BMI was 21.5 (range, 16-31), and the mean implant volume used was 300 cc (range, 95-475 cc). The mean follow-up duration was 18 months (range, 3-33 months). Additional patient and implant demographics are presented in Table 1.

**Table 1.** Patient Demographics, and Surgery/Implant Characteristics

Variable	Mean (range)
Age (years)	37 (18-63)
BMI (kg/m^2^)	21.5 (16-31)
Primary breast augmentation	313
Primary mastopexy–augmentation	17
Implant volume (cc)	300 (95-475)
Implant type	
Round	160
Ergonomix	180
Ergonomix2	320

The overall complication rate was 0.9%, Table 2. One acute complication was recorded in a mastopexy–augmentation case, where the patient experienced active bleeding from the vertical mastopexy incision on postoperative Day 1. No other early-onset complication was observed.

**Table 2.** Key Complications

	Complications	Rate, % (*n*)
Acute	Bleeding	0.3 (1)
	Seroma	0
	Wound breakdown	0
	Infections	0
Follow-up visits	Pain	0
	Capsular contracture (Baker grade III–IV)	0
	Implant lateralization	0
	Symmastia	0
	Inferior malposition	0
	High-riding implant	0.6 (2)
Overall		0.9 (3)

**Figure 5.**
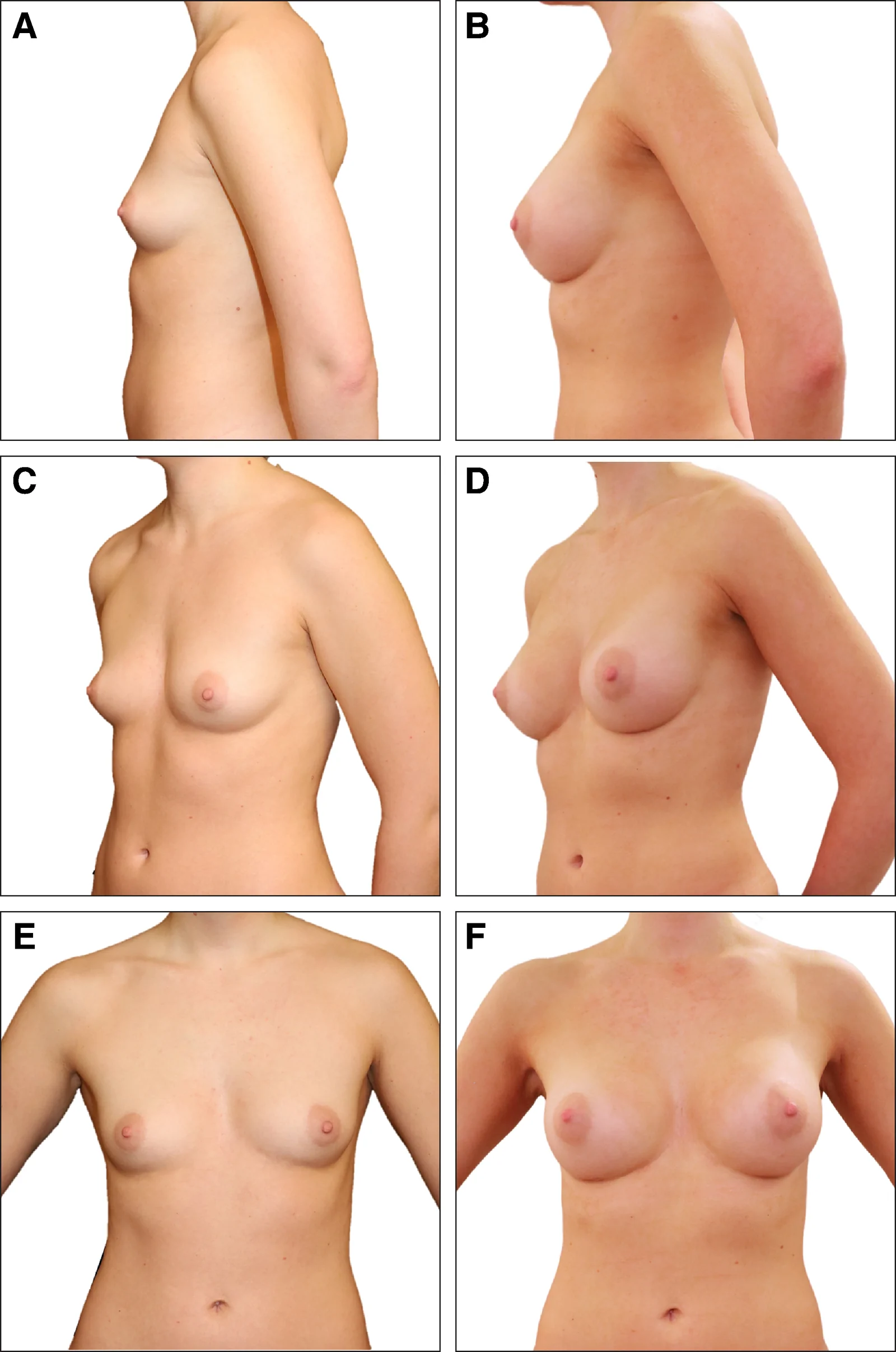
(A, C, E) Preoperative and (B, D, F) 2-year postoperative results of a 25-year-old female patient following Tissue-Preserving Breast Augmentation with E2SM-250Q

**Figure 6.**
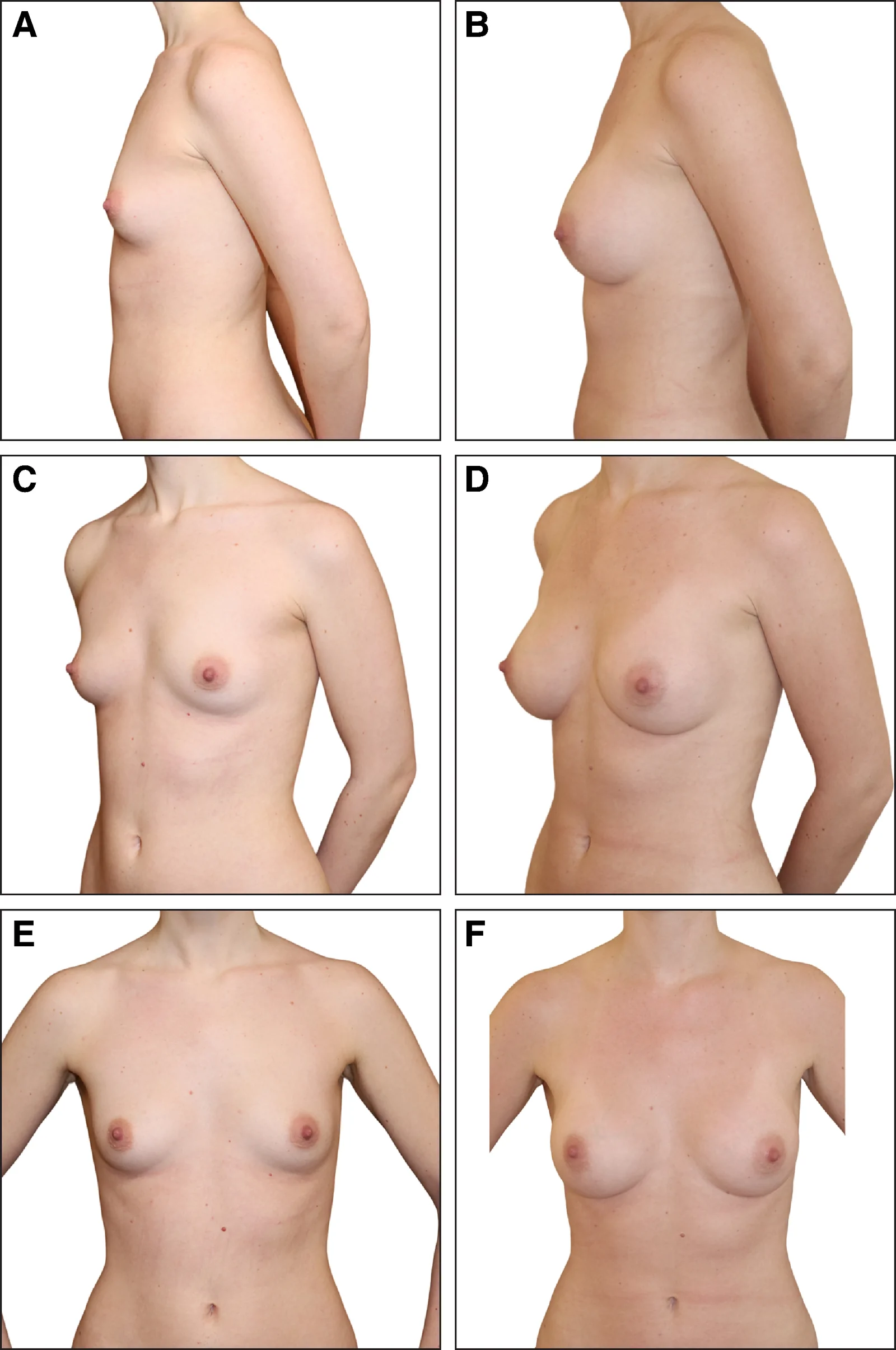
(A, C, E) Preoperative and (B, D, F) 1-year postoperative results of a 26-year-old female patient following Tissue-Preserving Breast Augmentation with E2SM-260Q.

**Figure 7.**
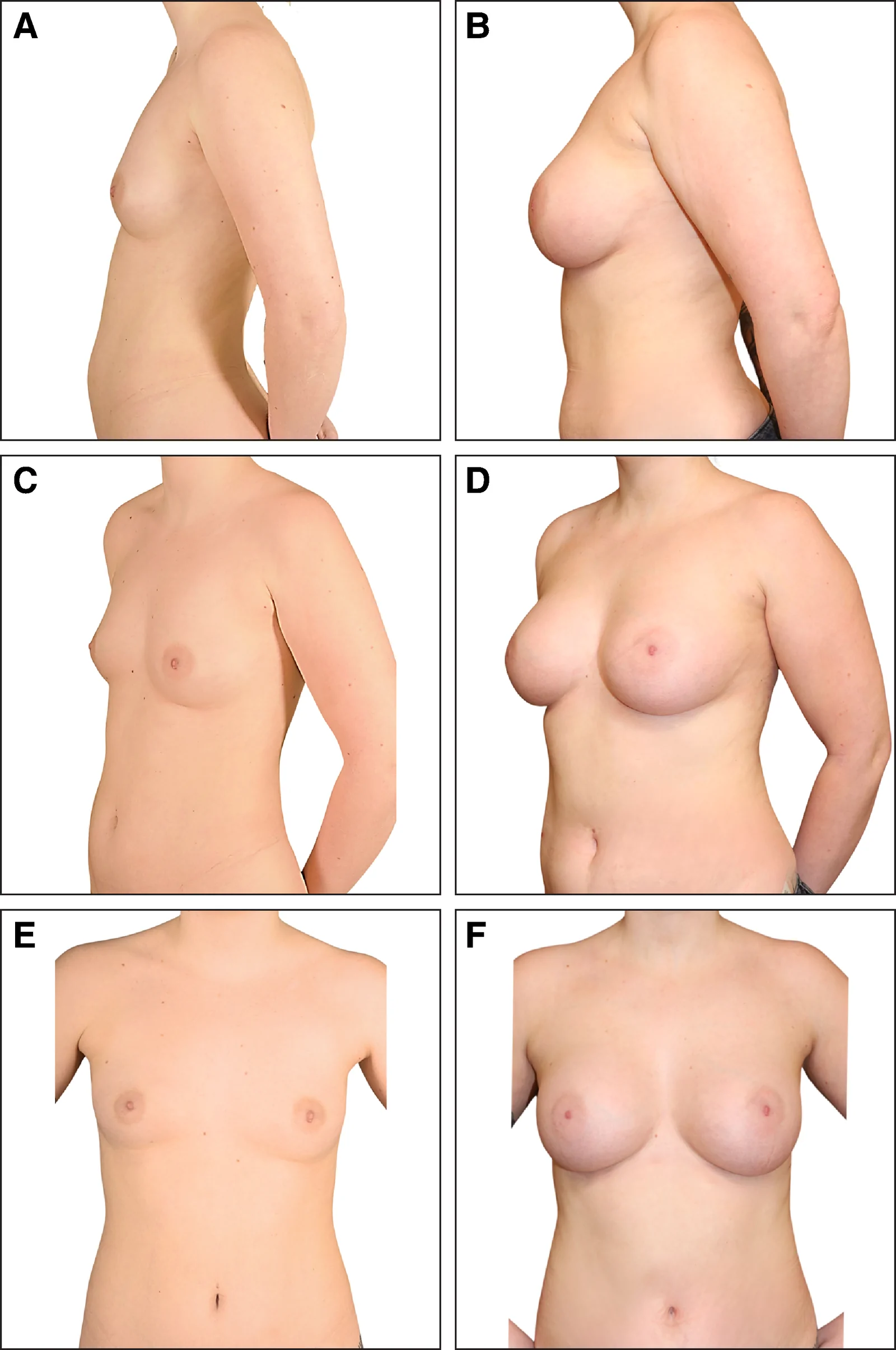
(A, C, E) Preoperative and (B, D, F) 2-year postoperative results of a 24-year-old female patient following Tissue-Preserving Breast Augmentation with E2SD-360Q. Performed by the senior author C.R.

**Figure 8.**
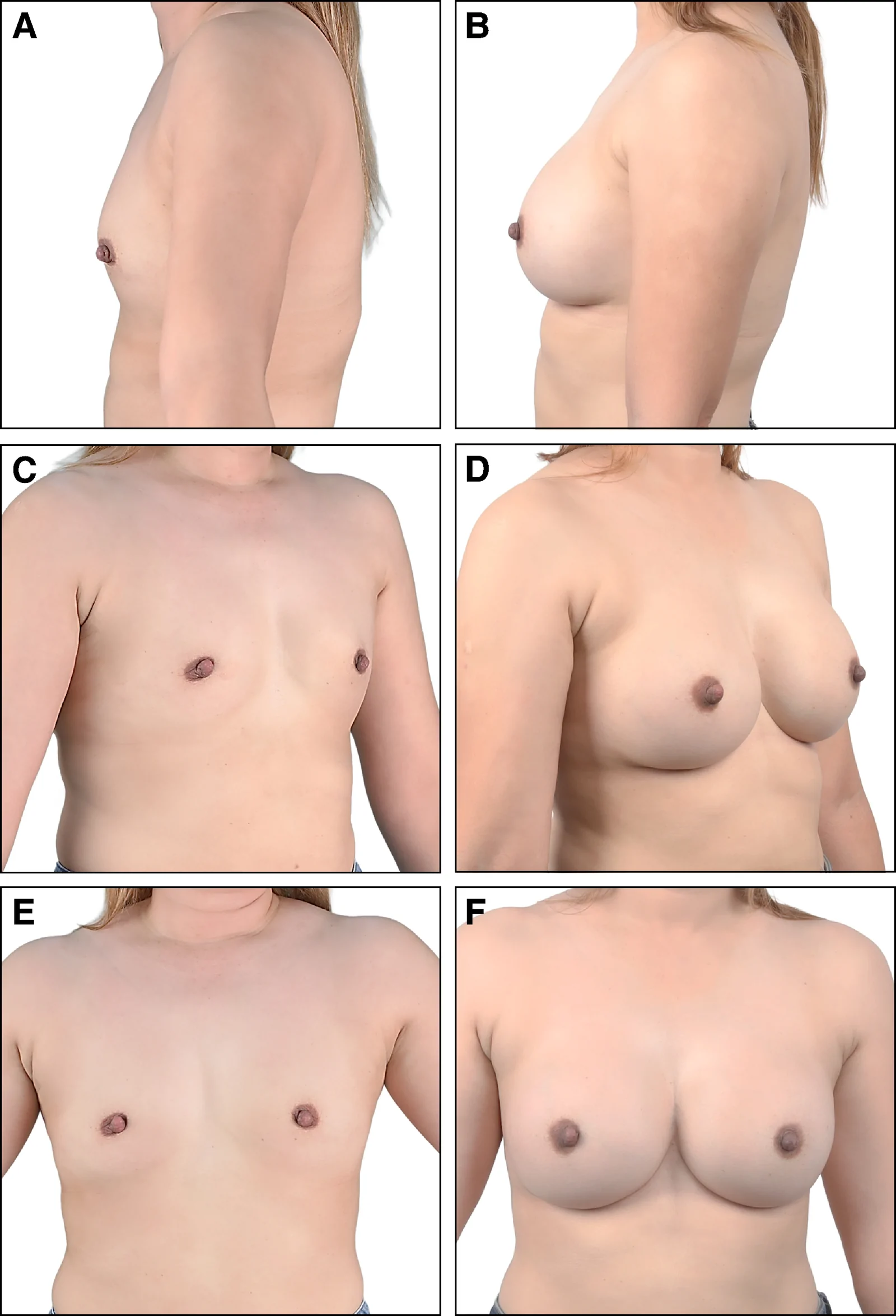
(A, C, E) Preoperative and (B, D, F) 2-year postoperative results of a 37-year-old female patient following Tissue-Preserving Breast Augmentation with RSF-375. Performed by the senior author A.W.

During follow-up visits at 2 weeks, and ultrasound-assisted follow-ups at 6, 12, and 24 months, no cases of capsular contracture, rupture, implant lateralization, symmastia, or inferior or lateral malposition were detected. However, 3 patients presented with visible rippling in the lateral and medial breast poles. Additionally, 2 patients underwent surgical correction because of a unilateral high-riding implant identified 1 year postoperatively. Outcomes at 6 months, 1 year, and 2 years postoperatively are presented in Figures 5-8.

## DISCUSSION

Historically, breast augmentation evolved from early subglandular techniques toward submuscular and dual-plane approaches because of concerns regarding capsular contracture and implant stability. Advances in implant surface technology, shell design, gel cohesivity, and surgical technique have since enabled a reconsideration of prepectoral placement in appropriately selected patients.

The concept of TPBA, performed through an IMF incision, aims to provide surgeons with an alternative, standardized, and minimally invasive approach to breast augmentation. This technique prioritizes preservation of the SFS and the breast's parenchymal support system. TPBA relies on bolus hydrodissection, blunt channel creation, and balloon-assisted prepectoral pocket dissection, performed on top of the PMM fascia.

By creating a tight pocket while maintaining the integrity of the internal breast-supporting structures, the method offers enhanced implant stability and reduces the risk of malposition. Hydrodissection has been widely validated for its effectiveness in separating tissue planes while preserving critical anatomical components such as nerves, blood vessels, and the lymphatic system, thereby minimizing complications, including bleeding or unintended trauma.^31,32^ In addition to its anatomical advantages, this approach is associated with reduced operative times, quicker recovery, and improved postoperative comfort. The implant is placed prepectoral, on top of the PMM fascia. This location is optimal, presenting a relatively avascular space with clear anatomical borders and avoiding the risk of accidentally placing the implant intraglandular. Consequently, the bolus hydrodissection and blunt balloon expansion, in this space, ensure optimal conditions for a bloodless dissection.

The evolution of minimally invasive surgical techniques has transformed multiple medical fields. In orthopedic surgery, arthroscopic approaches have largely replaced open procedures, reducing recovery time, postoperative pain, and surgical trauma.^33^ Similarly, laparoscopic techniques in general surgery have decreased tissue disruption and complication rates and improved patient satisfaction.^34^ These paradigm shifts reflect a broader movement toward surgical techniques that prioritize anatomical preservation, functional outcomes, and patient-centered care.

TPBA represents a logical extension of this philosophy in aesthetic breast surgery. By preserving internal breast structures and minimizing tissue trauma through bolus hydrodissection and blunt, balloon-assisted pocket creation, TPBA aligns with principles proven successful across other surgical disciplines, enhancing both clinical outcomes and patient experience.

Although blunt pocket creation is not novel in itself, the technique described here differs from previously reported approaches by combining bolus hydrodissection with balloon-assisted blunt dissection within a narrowly defined, standardized prepectoral channel. This approach allows controlled, reproducible pocket geometry while minimizing tissue trauma. Unlike sweeping or tearing maneuvers, balloon expansion develops the prepectoral plane evenly along natural anatomical boundaries, typically resulting in a near-bloodless pocket with limited need for hemostasis.

Although balloon-assisted dissection could theoretically be performed in the submuscular plane, subpectoral and dual-plane approaches inherently involve muscle manipulation and exposure to dynamic muscular forces. These factors may affect implant stability and are associated with risks such as animation deformity, implant displacement, and postoperative discomfort.

The standardized prepectoral, tissue-preserving approach described in this study avoids direct muscle involvement while maintaining precise pocket control.

In this study, implant sizes ranged from 95 to 475 cc. The use of smaller-volume implants in the prepectoral plane appears to contribute significantly to favorable aesthetic outcomes by providing a volumetric enhancement without the complications typically associated with larger implants. Smaller implants decrease mechanical stress on the breast's supportive tissues, thereby reducing the risk of malposition, tissue thinning, and progressive ptosis.^35^

Definitive implant size limitations for this technique cannot be established, as long-term breast stability is multifactorial and patient-specific, depending on factors such as skin quality, connective tissue integrity, biological age, hormonal influences, weight fluctuations, and pregnancy history.

Based on available anatomical studies and our clinical experience, implant width in this series was limited to the approximate diameter of the circummammary ligament (10-12 cm). Within this range, implant widths of up to 12 cm appear compatible with preservation of intrinsic suspensory structures and long-term stability. Exceeding this width may increase the risk of overstretching or disrupting the circummammary ligament and associated stabilizing elements. This threshold, however, requires validation through larger cohorts and longer-term follow-up studies.

### Incision Length

In this study, the minimum incision length applied was 2.5 cm, as shorter incisions limited the ability to introduce the retractor or adequately assess the pocket with the surgeon's index finger. Round and Ergonomix implants require slightly longer incisions because of their shell configuration, compared with Ergonomix2 implants. The length of the incision also depends on the technique and strength of the surgeon utilizing the funnel-assisted implantation. As a guideline for the length of the incision, the following formula was used and found to reduce the risk of implant shell damage or rupture during implantation: implant volume (cc) ÷ 10 = incision length (cm). For example, a 350 cc implant requires an incision length of 3.5 cm.

### Outcomes and Observations

In this series, no acute complications, such as seroma, hematoma, or infection, were observed in relation to the TPBA procedure itself. One case of postoperative bleeding was noted at the vertical mastopexy incision line. Although the follow-up period remained relatively short, the absence of early displacement complications is encouraging. Most malposition issues typically arise within the first year, although late-onset complications remain a consideration.^36^

No cases of lateralization, symmastia, or inferior malposition were observed. Two patients developed unilateral high-riding implants, which were surgically corrected. These findings support the effectiveness of TPBA and underscore the value of preserving the breast's natural support system. They also highlight the importance of meticulous surgical planning, precise pocket dissection, and accurate implant placement.

During the initial phase of adopting the TPBA technique, a 2 cm pinch thickness in the upper medial quadrant was used. This was subsequently reduced to 1 cm, based on the assumption that rippling would not occur, even in patients with very thin skin. However, 3 cases of rippling were subsequently detected, prompting a reevaluation of the approach. Consequently, the 2 cm pinch thickness in the upper medial quadrant was reinstated and is now recommended as a guideline for minimizing long-term risks such as rippling and implant visibility.

Reported complication rates following breast augmentation vary widely in the literature and depend on implant type, pocket location, surgical technique, and duration of follow-up. In our practice, baseline complication rates before the introduction of TPBA were already low and comparable to those reported in the literature. TPBA was therefore not developed to reduce complication rates, and its introduction did not result in a statistically meaningful reduction in complications. Rather, TPBA is presented as an additional surgical option that may offer advantages in selected patients with respect to tissue preservation, postoperative comfort, and functional recovery.

The TPBA technique has a relatively short learning curve for board-certified plastic surgeons familiar with breast anatomy and prepectoral dissection. The procedural steps are intuitive, and the use of balloon-assisted expansion facilitates controlled and reproducible pocket development.

Careful attention is required during pocket creation, as the inflatable balloon follows the path of least resistance. During expansion, the surgeon must actively maintain the balloon in a central position posterior to the NAC. Inadequate control may result in excessive lateral pocket expansion or a high-riding implant. Meticulous adherence to anatomical landmarks is therefore essential to achieve optimal implant positioning.

### Role of Anesthesia and Patient Appeal

The TPBA technique presented in this paper can be safely performed under local anesthesia, with or without light sedation, thereby eliminating general anesthesia-related risks.^37^ This approach addressed a common concern among patients, the fear of not waking up after surgery, and potentially broadened the appeal of breast augmentation to a new cohort of women, who previously might have declined a breast augmentation because of anesthesia-related apprehensions.^38^

### Patient Selection and Limitations

The TPBA technique is most suitable for patients with an intact internal breast stabilization system and sufficient soft tissue coverage. Patients with compromised connective tissue, resulting from factors such as significant weight loss, pseudoptosis without pregnancy, Ehlers–Danlos syndrome, severe striae, or upper pole tissue thickness of <2 cm, are considered suboptimal candidates. In such cases, complementary procedures such as autologous fat grafting (ie, hybrid augmentation) are advised. Alternatively, a conventional submuscular or dual-plane approach may remain the preferred technique for certain anatomies. However, patients with upper pole tissue thickness of <2 cm but firm connective tissue and subcutaneous coverage and thicker skin may still be suitable candidates for the TPBA technique.

### Limitations

This study is limited by the timeframe for postoperative assessment. The reported outcomes included evaluations conducted between 2 weeks, 6 months, and 24 months following surgery, with a mean follow-up period of 18 months. Additionally, the use of a single implant manufacturer is also a limitation. Future long-term studies incorporating different implant types and manufacturers would be valuable in assessing the sustained outcomes of this technique, including aesthetic results, patient satisfaction, and complication rates.

## CONCLUSIONS

TPBA is a safe, reproducible, and reliable technique for breast augmentation through the IMF. It offers a short learning curve, a low risk of surgical complications, and can be performed under local anesthesia with or without sedation, facilitating rapid recovery. The authors believe that this innovative and standardized technique represents a new option and viable alternative for breast augmentation, offering high levels of satisfaction for both patients and surgeons.

## Supplementary Material

sjag038_Supplementary_Data
